# The impact of pre-operative axial length on myopic shift 3 years after congenital and developmental cataract surgery and intraocular lens implantation

**DOI:** 10.3389/fmed.2022.1093276

**Published:** 2023-01-11

**Authors:** Xiyue Zhou, Fan Fan, Xin Liu, Jianing Yang, Tianke Yang, Yi Luo

**Affiliations:** Department of Ophthalmology, Eye Institute, Eye and ENT Hospital, Fudan University, Shanghai, China

**Keywords:** myopic shift, pre-operative axial length, congenital/developmental cataract, cataract surgery, IOL implantation

## Abstract

**Purpose:**

To investigate the impact of the pre-operative axial length (AL) on myopic shift (MS) 3 years after primary intraocular lens (IOL) implantation in congenital/developmental cataract patients.

**Methods:**

A retrospective study of patients who underwent congenital/developmental cataract surgery and primary IOL implantation at age 2–3 years at EENT Hospital was conducted. All patients were followed up regularly for at least 3 years after surgery. Refractive outcomes, including spherical equivalent (SE) and MS, were collected at each follow-up.

**Results:**

Forty eyes from 40 patients were included. The mean age at surgery was 2.56 ± 0.57 years old, and the mean follow-up time was 3.05 ± 0.22 years. Patients were divided into two groups: Group 1 included 20 patients with longer pre-operative ALs (≥22 mm), and Group 2 included 20 patients with average pre-operative ALs (<22 mm). By the last follow-up, the MS was 2.13 (0.38, 2.63) D in Group 1 and 3.88 (2.85, 5.72) D in Group 2. The post-operative MS in Group 2 was statistically greater than that in Group 1 at 3 years after surgery (*P* < 0.001).

**Conclusion:**

In congenital/developmental cataract patients who underwent cataract extraction and primary IOL implantation at age 2–3 years, eyes with longer pre-operative ALs had a slower MS than those with average pre-operative ALs 3 years after surgery. This finding could have implications for the target refraction decision in congenital/developmental cataract surgery.

## Introduction

Myopic shift (MS) occurs after congenital/developmental cataract extraction and intraocular lens (IOL) implantation in most children. MS usually increases with age, and the rate of MS is most rapid during the first 1.5 years of life (1). Considering that MS increases with age, most clinicians choose an under corrected target diopter when performing IOL implantation in children (2–5).

Due to the rapid growth of children’s eyeballs and the great individual differences in children with congenital/developmental cataracts, MS can be quite unpredictable in childhood and varies substantially among patients, which can lead to large refractive errors or even high myopia. Although target diopter selection takes age into consideration, many children still require additional refractive correction within a few years, and even IOL replacement is needed in some situations to correct anisometropia and improve visual acuity (6–8). Therefore, other factors related to MS should be taken into account when selecting the target diopter, and pre-operative axial length (AL) is quite important.

An increasing number of congenital/developmental cataract patients are being diagnosed with myopia before surgery or have a longer pre-operative AL than average. However, there has been little previous research on the impact of the pre-operative AL on post-operative MS.

In this study, we reviewed the visual and refractive outcomes of children with different ALs who underwent congenital/developmental cataract extraction and primary IOL implantation at 2–3 years old. Additionally, the impact of the pre-operative AL on MS was investigated 3 years after primary IOL implantation in congenital/developmental cataract patients.

## Materials and methods

### Ethic declaration

The Institutional Review Board of the Eye and ENT Hospital of Fudan University, Shanghai, China, approved this retrospective cohort study. All procedures were conducted in agreement with the tenets of the Declaration of Helsinki. Written consent forms were signed by the guardians of the patients for the use of their medical data for research purposes before surgery.

### Study population

This retrospective cohort study was performed on the medical records of children who underwent congenital/developmental cataract surgery at Eye and ENT Hospital of Fudan University between 2014 and 2018. The age at diagnosis and surgery had to be 2–3 years old, and the patients were followed up regularly for at least 3 years after surgery. Diseased eyes were included in children with unilateral congenital/developmental cataract, and only right eyes were included in children with bilateral congenital/developmental cataract.

The exclusion criteria were other eye diseases, such as severe posterior and combined persistent fetal vasculature (9), congenital microcornea, congenital iris defects and congenital glaucoma; systemic diseases, such as cerebral palsy and congenital heart disease; serious post-operative complications, such as uveitis; and failure to follow regular follow-up or amblyopia training.

### Surgical technique

All surgeries were performed by the same experienced surgeon. Lensectomy, anterior vitrectomy and primary IOL implantation were performed, and the IOLs were implanted in the capsular bag. The IOL power was calculated on the basis of the SRK/T formula and the Hoffer Q formula. For patients who failed to cooperate with the keratometry measurement, we used an average keratometry (45 D for 2 year olds, 44 D for 2.5 year olds, 43.5 D for 3 year olds) when calculating the IOL power (10, 11). The target diopter was (7-age) D, targeting an hyperopia under correction based on the age at surgery.

### Post-operative assessment

All patients were referred to the same pediatric amblyopia expert within one week after the operation. They had optometric assessments, lenses with best refractive correction and individualized amblyopia training plans. Follow-up was performed 1 day, 1 week, 1 month and every 4 months after surgery for at least 3 years. During the follow-up, all patients kept the individualized amblyopia training plan and changed the glasses when necessary. Post-operative complications, adherence to amblyopia training, and visual and refractive outcomes were collected at each follow-up. Children with unilateral congenital/developmental cataract had an extra covering on the contralateral eye every day, lasting 3–6 h according to the follow-up age and the amblyopia severity (12–15).

### Statistical analysis

Statistical analyses were performed using IBM SPSS statistics (version 25.0; IBM Corp., Somers, NY, USA). Descriptive statistics were used to analyse the demographics and clinical characteristics of the population. Continuous variables are presented as the mean ± standard or median (quartile). Independent-samples *t*-tests were used to compare the means. Mann–Whitney U test was applied to compare the medians. The linear regression model was used to analysis the impact of pre-operative AL on MS. A *P*-value < 0.05 was considered statistically significant difference.

## Results

### Basic characteristics

From 2014 to 2018, a total of 98 congenital/developmental cataract patients treated by Dr. Luo’s team underwent primary IOL implantation at 2–3 years of age at Eye and ENT Hospital of Fudan University. Of the 98 patients, we included 40 patients who met the inclusion criteria. Twenty-one (52.5%) had bilateral cataracts, and nineteen (47.5%) had unilateral cataracts. The percentage of males to females was 22 (55.0%) versus 18 (45.0%). The mean age at surgery was 2.56 ± 0.57 years old. The mean follow-up time was 3.05 ± 0.22 years.

The patients were divided into two groups according to the pre-operative AL. Group 1 included 20 patients with longer pre-operative ALs (≥22 mm), and Group 2 included 20 patients with average pre-operative ALs (<22 mm). The mean pre-operative AL was 23.17 ± 0.81 mm in Group 1 and 20.85 ± 0.69 mm in Group 2. Table 1 shows the characteristics of the two groups. There was no significant difference in the follow-up time (*P* = 0.095) or basic characteristics except for AL.

**TABLE 1:** Characteristics of the two groups.

	Group 1	Group 2	*P*-value
Eyes (*n*)	20	20	–
Male/female (*n*)	13/7	9/11	0.204
Unilateral/bilateral (eyes)	8/12	11/9	0.342
**Cataract morphology (eyes)**			
Total white	2	2	–
Nuclear	1	1	–
Cortical	4	1	–
Posterior subcapsular	1	6	–
Mixed	2	4	–
Others*	10	6	–
Age at surgery (y)	2.53 ± 0.60	2.59 ± 0.56	0.737
Pre-operative AL (mm)	23.17 ± 0.81	20.85 ± 0.69	<0.001*
Follow-up time (y)	3.00 ± 0.15	3.11 ± 0.26	0.095
Age at the last follow-up (y)	5.53 ± 0.61	5.70 ± 0.60	0.362

*n*, number of patients; y, years.

*P < 0.05. *Others include point-like, perinuclear, lamellar, anterior polar, and other opacity.

### Comparison of MS between the two different AL groups

**FIGURE 1:**
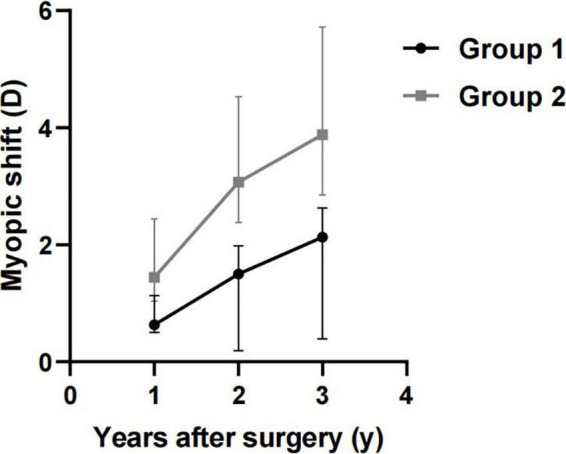
Comparison of myopic shift (MS) at 1–3 years after surgery in Group 1 and Group 2.

Table 2 shows the refractive outcomes of the two groups. One year after surgery, the MS of Group 1 and Group 2 was significantly different [0.63 (0.5, 1.13) D vs. 1.44 (1.04, 2.44) D, *P* < 0.001]. The MS at two years after surgery was 1.50 (0.19, 1.98) D in Group 1 and 3.07 (2.38, 4.53) D in Group 2. By the time of last follow-up (3 years after surgery), the MS of Group 1 and Group 2 were 2.13 (0.38, 2.63) D and 3.88 (2.85, 5.72) D, respectively. The post-operative MS in Group 2 was statistically greater than that in Group 1 at two and three years after surgery (*P* < 0.001). Figure 1 shows the comparison of MS at 1–3 years after surgery between Group 1 and Group 2.

**TABLE 2:** Refractive outcomes of the two groups.

	Group 1	Group 2	*P*-value
Eyes (*n*)	20	20	–
Initial SE (D)	3.59 ± 2.67	3.76 (3.06, 5.00)	0.569
SE at last follow-up (D)	2.29 ± 2.99	−0.01 ± 2.59	0.009*
MS 1 year after surgery (D)	0.63 (0.5, 1.13)	1.44 (1.04, 2.44)	<0.001*
MS 2 years after surgery (D)	1.50 (0.19, 1.98)	3.07 (2.38, 4.53)	<0.001*
MS 3 years after surgery (D)	2.13 (0.38, 2.63)	3.88 (2.85, 5.72)	<0.001*

*n*, number of patients; D, diopter; SE, spherical equivalent.

*P < 0.05.

### Comparison of MS between the unilateral and bilateral cases

**FIGURE 2:**
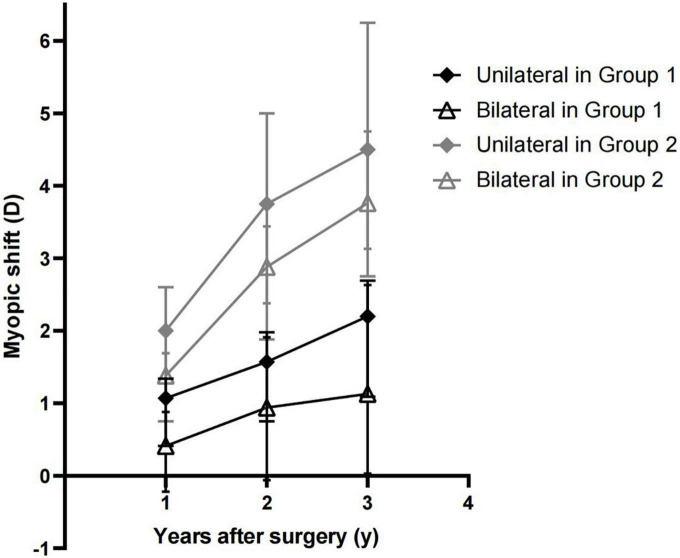
Comparison of myopic shift (MS) in patients with unilateral and bilateral cataracts in the same pre-operative axial length (AL) group.

Table 3 and Figure 2 present the comparison of MS between unilateral and bilateral congenital/developmental cataracts in the same group of pre-operative AL. No statistically significant difference was found in MS between unilateral and bilateral cataracts in the same group of pre-operative AL (*P* > 0.05).

**TABLE 3:** Comparison of myopic shift (MS) in patients with unilateral and bilateral cataracts in the same pre-operative axial length (AL) group.

	Group 1		*P*-value	Group 2		*P*-value
	Unilateral	Bilateral		Unilateral	Bilateral	
Eyes (*n*)	8	12	–	9	11	–
MS 1 year after surgery (D)	1.07 (0.41, 1.34)	0.41 (−0.22, 0.88)	0.054	2.00 (0.75, 2.60)	1.38 (1.07, 1.69)	0.304
MS 2 years after surgery (D)	1.57 (0.75, 1.98)	0.94 (−0.06, 1.91)	0.316	3.75 (1.88, 5.00)	2.88 (2.38, 3.44)	0.424
MS 3 years after surgery (D)	2.20 (1.09, 2.69)	1.13 (0.03, 2.63)	0.279	4.50 (2.75, 6.25)	3.76 (2.69, 4.75)	0.595

### The impact of pre-operative AL on MS

The linear regression model was used to analysis the impact of pre-operative AL on MS, with MS 3 years after surgery as the outcome variable and pre-operative AL as the main predictor. The confounding factors included laterality, gender and type of cataract.

The linear regression model turned out to be MS = 20.60 –0.76*pre-operational AL (R^2^ = 0.337, F = 4.445, *P* = 0.005). As the linear regression model showed, the impact of pre-operational AL on MS was statistically significant (b = −0.76, *t* = 2.80, *P* = 0.008). However, the impact of laterality, gender and type of cataract on MS was not statistically significant (*P* = 0.136, 0.334, and 0.108).

## Discussion

There is little previous research on the impact of the pre-operative AL on post-operative MS. An earlier retrospective study conducted in Peru (16) found no statistically significant relationship between the initial AL and MS 3 years after IOL implantation. The MS was 3.2 ± 3.3 D in children with longer ALs and 3.9 ± 3.2 D in those with shorter ALs (*P* = 0.359). This earlier study included congenital cataracts in children younger than 4 years old and chose an AL of 21.5 mm as the group division. As numerous studies have observed, the growth of AL was the most significant during the first 2 years of life and tended to be stable as age increased (17–21). Therefore, the mixing of children under 12 months with children aged 3–4 years old in the earlier study may have led to deviation of the results. In our study, we only included patients aged 2–3 years old, which not only captured the period of greater ocular growth but also controlled the impact of operative age on post-operative MS.

With reference to a 3-year-old Asian cohort (349 children) and another study in 4,350 Chinese children aged 3 to 4 years, which found average ALs of 21.73 ± 0.66 mm (95% CI: 21.6–21.80 mm) (22) and 22.10 ± 0.79 mm (95% CI: 20.55 –23.65 mm) (23), we chose an AL of 22 mm as the standard for grouping patients aged 2–3 years old in our study.

Myopic shift after congenital/developmental cataract surgery and IOL implantation is quite complicated and difficult to predict. The Eye and ENT Hospital of Fudan University is the largest tertiary referral center for pediatric cataracts in East China and receives almost all pediatric cataracts in this region (24). Based on the 3-year clinical data of congenital/developmental cataract patients with primary IOL implantation between 2 and 3 years old in our center, we observed that eyes with longer pre-operative ALs (≥22 mm) tended to have a slower MS than those with average pre-operative ALs (<22 mm) 3 years after surgery.

Axial length growth is associated with MS and myopia, (25) and a longer AL can be used to identify those at high risk of myopia in both pre-school and school-aged children (26, 27). However, in our study, a longer pre-operative AL led to a slower MS in children with congenital/developmental cataracts with pseudophakic eyes. The opposite result may be related to the impact of congenital/developmental cataracts on eyeball growth. Seven et al. (28) found a lower growth rate of AL in pseudophakic eyes than in phakic eyes, while Wilson et al. (18) found that eyes treated for monocular cataracts in infancy had axial growth similar to that of fellow eyes. This finding and the reasons behind it remain to be solved in further studies.

Compared with that of bilateral congenital/developmental cataract patients, the prognosis of unilateral congenital/developmental cataract patients is usually worse (29–31). Earlier studies have already found that children with unilateral congenital cataracts had greater MS after surgery (20, 32). Additionally, studies have shown that the growth of AL in unilateral patients tends to be greater than that in bilateral patients (33). Children with unilateral congenital cataracts are more likely to have high myopia and great anisometropia in the long term after the operation, (28) the mean of which can be up to −3.50 D (−19.63 D–+2.75 D) at the age of five. In our study, the MS in unilateral congenital cataracts was numerically larger, but no statistically significant difference was found in MS between unilateral and bilateral congenital/developmental cataracts of each group. The results of the linear regression model also showed that there was no statistical relevance between laterality and MS (*P* = 0.136). In our study, all unilateral congenital/developmental cataract patients had an extra covering on the contralateral eye every day, which may have reduced the differences in MS between children with unilateral and bilateral congenital/developmental cataracts.

Keratometry is another refractive-related biometry characteristic. It has been widely acknowledged that infants have a steeper corneal curvature than older children. A study showed a linear decline in mean keratometry during the first 6 months of life, while no significant change was found in the keratometry value with increasing age beyond 6 months (10). That is, the corneal curvature decreased with age and stabilized after 6 months of age (34). With this growth pattern of keratometry, patients aged 2–3 years old would have a relatively stable keratometry at surgery, and keratometry and its influence on post-operative MS could be insignificant or minimally significant in our study. Thus, for patients who failed to cooperate with the examination, an average keratometry from those of the same age was used to calculate the IOL power.

To the best of our knowledge, our study is the first to demonstrate the impact of the pre-operative AL on MS in children with congenital/developmental cataracts. As the age of the global myopia population decreases, this result can help to guide the prediction and control of MS in congenital/developmental cataract patients with longer ALs. However, our study still has its limitations. Long-term changes of MS cannot be observed during 3-year follow-up, as some patients with high and pathological myopia tend to grow even further. The number of participants was not large enough. We did not include the anterior chamber depth in the analysis, which was also an important variable in biometry measurement. Also, we were unable to measure the post-operative AL of all patients. Future studies for long-term outcomes will enlarge the sample size, incorporate anterior chamber depth and post-operative AL changes.

In conclusion, in congenital/developmental cataract patients who underwent surgery at 2–3 years old, eyes with longer pre-operative ALs had a slower MS than those with average pre-operative ALs. This finding could have implications for the target refraction decision in congenital/developmental cataract surgery. Accurate prediction of post-operative MS in congenital/developmental cataract patients remains challenging. Studies on MS and the pre-operative factors influencing it, such as post-operative AL, will be helpful to better predict and control MS after congenital/developmental cataract surgery and can help clinicians to make optimal treatment decisions.

## Data availability statement

The raw data supporting the conclusions of this article will be made available by the authors, without undue reservation.

## Ethics statement

The studies involving human participants were reviewed and approved by the Institutional Review Board of the Eye and ENT Hospital of Fudan University, Shanghai, China. Written informed consent to participate in this study was provided by the participants or their legal guardian/next of kin.

## Author contributions

XZ was responsible for conception and design, analysis and interpretation of data, and writing the manuscript. FF was responsible for conception and design, analysis and interpretation of data, critical revision of the manuscript, and supervision. XL, JY, and TY were responsible for data collection. YL was responsible for conception and design, technical support, and critical revision of the manuscript. All authors contributed to the article and approved the submitted version.
